# FROG - Fingerprinting Genomic Variation Ontology

**DOI:** 10.1371/journal.pone.0134693

**Published:** 2015-08-05

**Authors:** E. Abinaya, Pankaj Narang, Anshu Bhardwaj

**Affiliations:** 1 Department of Bioinformatics, SASTRA University, Thanjavur, Tamil Nadu, India; 2 School of Computational and Integrative Sciences, Jawaharlal Nehru University, New Delhi, India; 3 Open Source Drug Discovery Unit, Council of Scientific and Industrial Research (CSIR), Anusandhan Bhawan, 2 Rafi Marg, New Delhi, 110001, India; Centre for Cellular and Molecular Biology, INDIA

## Abstract

Genetic variations play a crucial role in differential phenotypic outcomes. Given the complexity in establishing this correlation and the enormous data available today, it is imperative to design machine-readable, efficient methods to store, label, search and analyze this data. A semantic approach, FROG: “FingeRprinting Ontology of Genomic variations” is implemented to label variation data, based on its location, function and interactions. FROG has six levels to describe the variation annotation, namely, chromosome, DNA, RNA, protein, variations and interactions. Each level is a conceptual aggregation of logically connected attributes each of which comprises of various properties for the variant. For example, in chromosome level, one of the attributes is location of variation and which has two properties, allosomes or autosomes. Another attribute is variation kind which has four properties, namely, indel, deletion, insertion, substitution. Likewise, there are 48 attributes and 278 properties to capture the variation annotation across six levels. Each property is then assigned a bit score which in turn leads to generation of a binary fingerprint based on the combination of these properties (mostly taken from existing variation ontologies). FROG is a novel and unique method designed for the purpose of labeling the entire variation data generated till date for efficient storage, search and analysis. A web-based platform is designed as a test case for users to navigate sample datasets and generate fingerprints. The platform is available at http://ab-openlab.csir.res.in/frog.

## Introduction

Genomic variations have been studied extensively to understand their role in disease association and drug responses. Over decades, this variation data has been systematically curated in various databases like the Single Nucleotide Polymorphism Database (dbSNP), which is the largest repository of SNP’s containing 184 million entries with more than 64 million distinct variants for 55 organisms (February 2010) [1], 1000 Genomes Project that sequenced 1092 genomes and reports 38 million SNP’s, 1.4 million bi-allelic indels, 14,000 large deletions and 1500 copy number variations [2], dbVar with genomic structural variations for different organisms [3] and many more [4,5]. Likewise, there are many repositories that are designed with the objective of capturing data related to human diseases like the OMIM with information on ~14,000 genes [6] and GAD with over 130,000 records on human genetic association studies of complex diseases and disorders [7]. In this context, global efforts in form of centralized archives and platforms have also been developed to capture genotype-phenotype interaction studies like the dbGaP [5] and GWAS Central [4]. In addition to these, there are other categories of databases which include locus specific information and are valuable from context of collecting and curating accurate lists of mutations and associated details on specific genes [8]. There are many tools that predict the outcome of structural variations at functional level like SIFT [9], PolyPhen-2 [10], PHD-SNP [11] etc, that have been developed to facilitate understanding the role of genomic variation from context of potential phenotypic impact. It has also been widely understood that environment also plays a significant role in phenotype modulation, further complicating the genotype and phenotype correlation. More recently, the Human Longevity Inc. launched an initiative to sequence half-million to one million human genomes per year as the world’s largest sequencing effort to further the understanding of genotype-phenotype association (http://www.genengnews.com/gen-news-highlights/venter-s-new-goal-world-s-largest-sequencing-operation/81249577/). There is clearly a need to develop systems to take advantage of this multidimensional big data in establishing robust genotype to phenotype correlations.

Global collaborations and experts have established standards for variation data curation and exchange; however, they have met with limited acceptability and implementation. The Gen2Phen project [12] and more recently The Human Variome Project Consortium [13] aim to develop standards, systems and infrastructure that will allow the global genetic variation data to be harnessed in disease diagnosis and treatment. This is a humongous task given that the various databases on human genetic variation and diseases do not follow a standard, globally acceptable, ontology and format for data accessibility. Ontology is defined as a way to organize and formally conceptualize information in a knowledge domain with a controlled vocabulary having defined terms and relationships between them[14]. Gene Ontology (GO) [15] was the first biological ontology and has been widely used in data-intensive applications [16]. Similarly, there are attempts to develop ontology for genetic variation like the PAGE-OM [17] and VariO [14]. PAGE-OM describes a data structure for variation data description in form of object models. VariO is designed to capture the effects, consequences and mechanisms of variations using consistent terms but does not capture clinical data as PAGE-OM does.

Using the various ontology terms from PAGE-OM and VariO, herein, we proposed the fingerprinting methodology to label variation data, based on its location, function and interactions. Most of these properties were selected using ontology of variations such as PAGE-OM and VariO, and named it as FROG- “Fingerprinting ontology of genomic variations”. FROG includes 278 variation properties at six levels to map variation properties along with the phenotype data. Binary fingerprints (bit scores) have been designed to store each property. Each level, with multiple properties, is represented as binary fingerprints. Bit score identification of variation properties is a computationally efficient method to store and search these properties. In addition, FROG is also amenable for adding new properties and relationships and it is not limited by language barrier. The binary form of data storage and search is suitable for storing the enormous data that is being generated and will be released in public domain in future.

## Materials and Methods

### Variation Terms and Binary Codes

FROG has been conceptualized as a flexible and extensible system for which each variation term (property) is represented using binary codes. Most of the terms captured in FROG are adopted from the existing ontologies, PAGE-OM and VariO, and some new terms were introduced (Table 1). Taking a top-down approach, the classification system in FROG starts with blocks (or levels), including, chromosome, DNA, RNA, protein, variation and interactions. Each level is then categorized into attributes and each attribute has a set of terms defining the variant properties. Each property is then binary coded in form of ‘0’ and ‘1’ and allows for combination and permutation for representing various properties within and across the levels. Each property, given the number of combinations it can have, is assigned a bit value. For example, in case of amino acid change, the variation can be synonymous or non-synonymous. This can be represented as ‘1’ for synonymous and ‘0’ for non-synonymous change. Thus, this information can be stored in one bit where a variation annotation can take a value of either ‘0’ or ‘1’. Another example is where two bits may be required in combination to represent the property, for e.g., Transition type is represented by two bits where each bit can have value ‘0’ or ‘1’ in combination such as 00 –A to G, 01 –G to A, 10 –C to T, 11 –T to C. Similarly, a combination of three bits are required to store 8 types of transversion such as 000– A to C, 001–A to T, 010– G to C, 100– G to T, 011– C to A, 101– C to G, 110– T to A, and 111– T to G. This approach generated a total of 102 bits for representing 278 properties of genomic variants across six levels.

**Table 1:** Properties in FROG and their source.

S.No.	Properties in FROG	VariO	PAGE-OM
1.	Clinical Data Referenced		✓
2.	Repeats and penetrance		
3.	Outcome of substitution on function		
4.	Accessibility	✓	
5	Synonymous/non synonymous change		
6.	Allosomes / autosomes		
7.	Works on whole genome	✓	
8.	Detailed description of Substitution		
9.	Copy number variation		
10.	Hap map data integration		
11.	Experimental details		✓
12.	Organism independent	✓	✓
13.	Epigenetic changes	✓	

### Development of FROG framework

To showcase the application of FROG fingerprints, data comprising of nearly 1,42,500 variations from 37 mitochondrial genes is used [18]. Next, FROG fingerprints of DNA, protein and variation levels were generated for all these genomic variations using in-house PERL script. The bit(s) are assigned a “*” if the information of corresponding variation property is not applicable for the variant or not available in the dataset. These fingerprints were stored in MySQL relational database system. To provide the front end, FROG framework was designed using Galaxy [19]. Galaxy is an open source tool that provides a way to integrate tools to make them more interactive. The wrappers of various tools to search database using FROG fingerprints of various levels were developed using PERL scripts and integrated with Galaxy using XML files. The Galaxy interface has three components, a tool list, a canvas to run applications and a history panel. The users may select search options from the tool list for which the detailed search page appears in canvas. On selecting the filters and executing search, the results appear in the history panel. Each user on accessing FROG initiates a personal session which is not viewable by other users who are simultaneously accessing the search interface. The users may register and create an account on FROG to keep track of their search history and earlier analysis.

## Results and Discussion

### Scope and Architecture of the proposed classification: The Semantics

**Fig 1:**
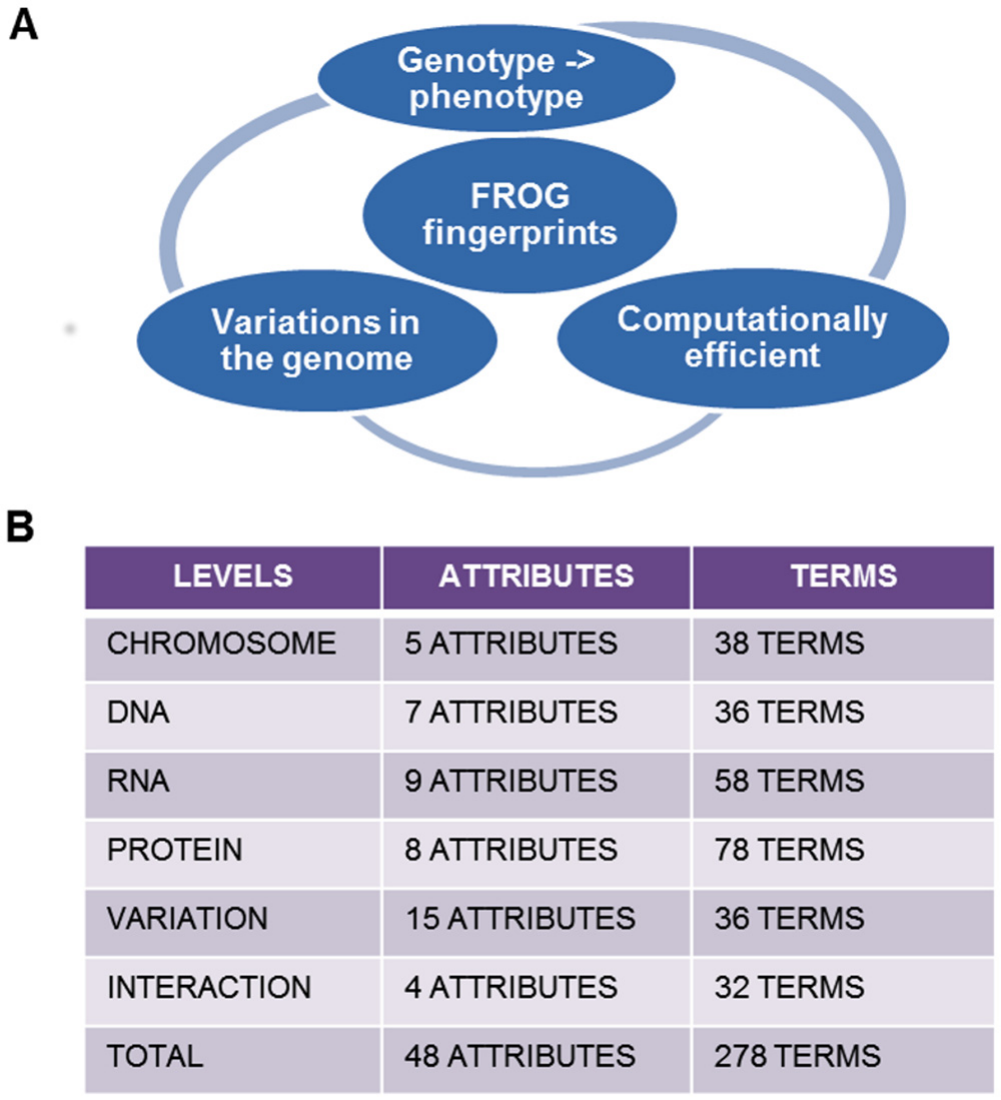
(A) Scope of FROG (B) Number of attributes and terms in six levels.

**Fig 2:**
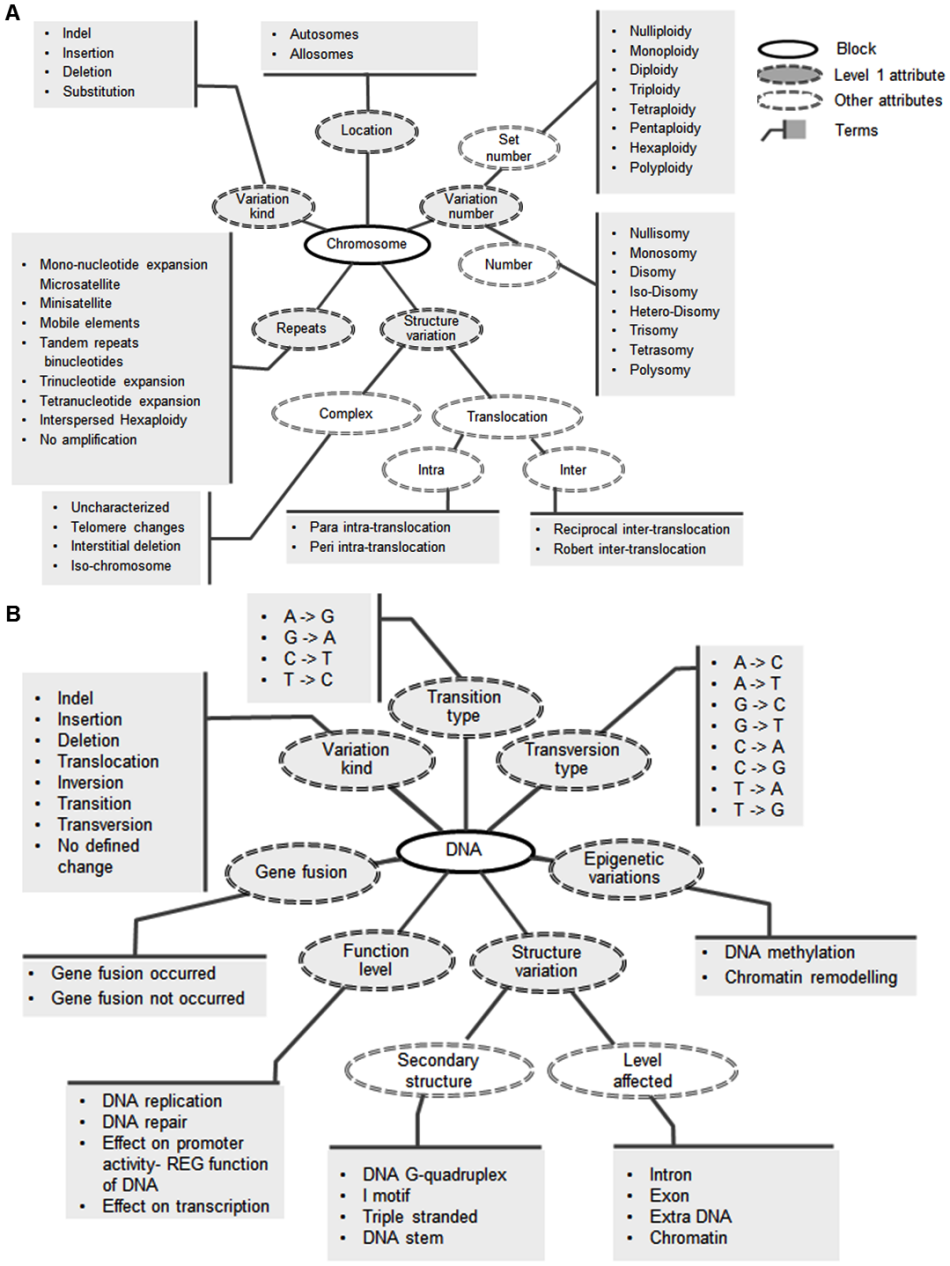
The attributes, ontology terms and their relationship within (A) Chromosome and (B) DNA level.

**Fig 3:**
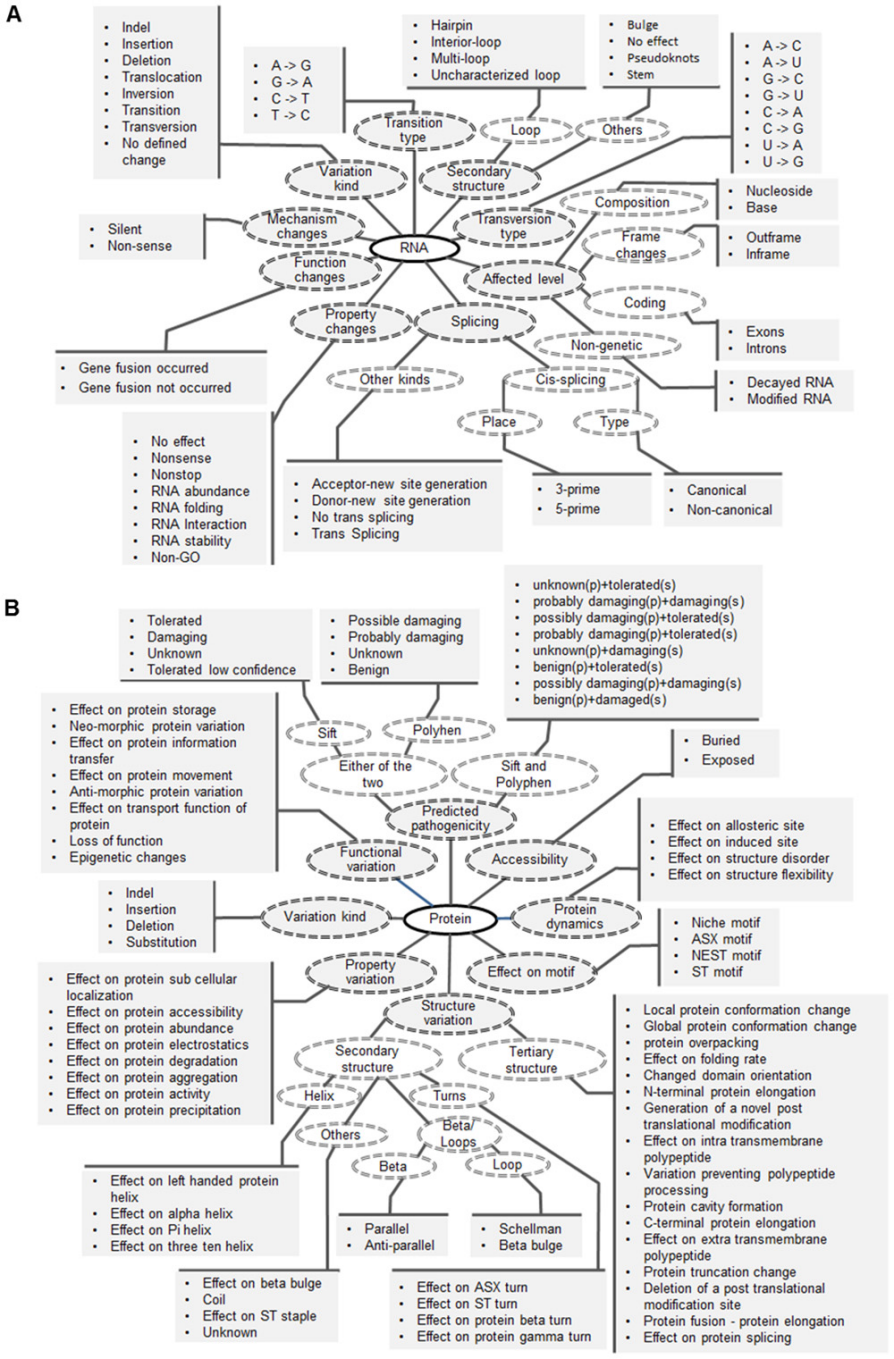
The attributes, ontology terms and their relationship within (A) RNA and (B) Protein level.

**Fig 4:**
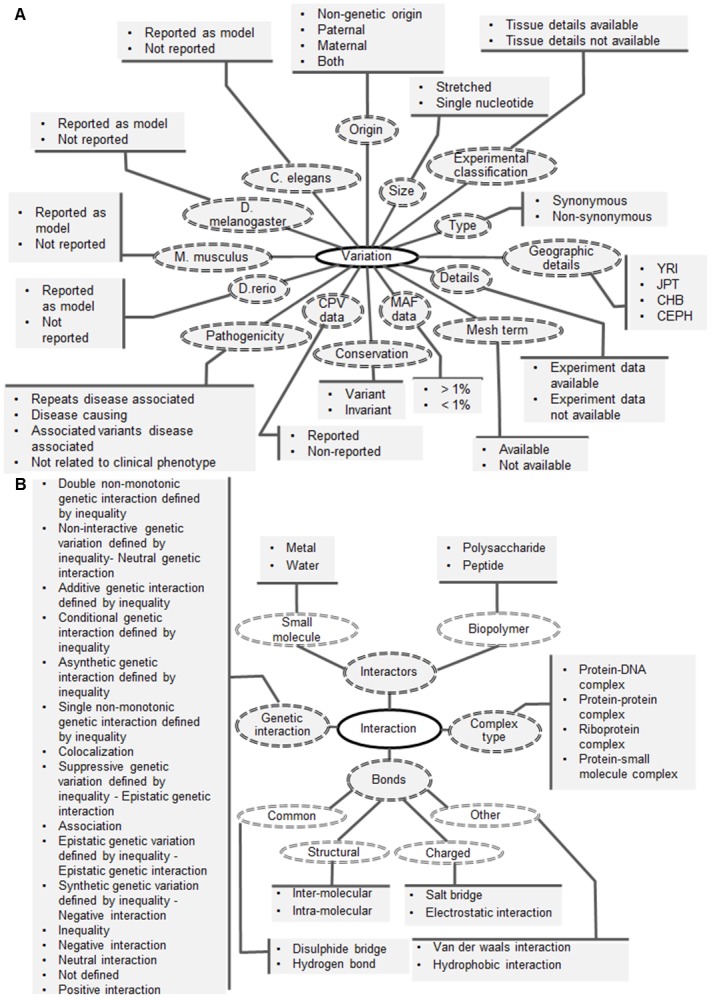
The attributes, ontology terms and their relationship within (A) Variation and (B) Interaction level.

FROG is an organism independent fingerprinting system designed to tag genomic variation at various levels of resolution (Fig 1A). FROG has six levels to describe the variation annotation. The six levels are Chromosome, DNA, RNA, Protein, Variations and Interactions (Fig 1B). Each level is a conceptual aggregation of logically connected attributes (Figs 2–4). For example, Chromosome level has five attributes, namely, location of variation (Allosomes or Autosomes), variation kind (indel, deletion, insertion, substitution), number of variations (diploidy, polyploidy—set number, disomy, polysomy—number of variation etc.), repeats (micro- and mini-satellite expansion, tandem repeats, interspersed repeats etc.) and structural variation (complex changes like telomere changes and translocations like inter and intra chromosomal) as shown in Fig 2A. Each attribute is a set describing various properties of the variant. In all there are 38 properties in the Chromosome level. Similarly, the other levels have a set of attributes to annotate the variation data.

In the DNA level there are seven attributes, namely, variation kind, transition, transversion, structural changes, functional changes, gene fusion and epigenetic changes (Fig 2B). Some of these attributes like transition and transversion are also defined as properties in the ‘variation kind’ attribute. The Transition attribute describes whether the change is pyrimidine to pyrimidine transition or purine to purine transition. Similarly, transversion describes all possible eight nucleotide changes, structural variation accounts for changes in secondary structure or extra chromosomal changes etc. The functional variation attribute includes effect on repair and replication, etc. Gene fusion and epigenetic changes (methylation and chromosome remodeling) are the two other attributes in DNA level to capture DNA variations that have been reported to be disease associated due to changes in secondary structure [20], epigenetic modifications [21] etc. There are a total of 36 properties in DNA level.

The RNA level includes nine attributes and 58 properties: variation kind (indel, transition, transversion inversion, translocation), Transition (pyrmidine to pyrimidine transition or purine to purine transition), Transversion (nucleotide changes), the secondary structure changes (loop changes—hairpin, interior loop, multiloop, complex structure changes—stem, bulge, pseudoknots), the levels affected (composition, frame (in or out), coding regions (introns or exons), splicing changes (cis- and trans- splicing), property changes (folding, stability, abundance etc.) and mechanism changes (silent and nonsense changes) as shown in Fig 3A.

The Protein level has eight attributes and 78 properties including the variation kind (indel, substitution, deletion, insertion), motif changes (niche motif, ST-motif, nest motif, ASX-motif), protein dynamics changes (allosteric site effect, structure disorder etc), structural changes (secondary and tertiary structure changes), functional changes (storage, gain or loss of function etc.), accessibility levels (buried or exposed), property changes (electrostatics, aggregation etc.) and predicted pathogenicity (predictions from PolyPhen and SIFT) as shown in Fig 3B.

The Variation and Interactions levels are common to the other four levels. The Variation level has 15 attributes and 36 properties, namely, variation origin (paternal, maternal, non-genetic and both), variation level (Chromosome, DNA, RNA, Protein), classification of variation (single base or amino acid change or a stretch of nucleotides or amino acids), association changes (synonymous or non-synonymous), copy number variation (reported or not), minor allele frequency (greater or less than 1%), experiment details (data available or not), tissue details (data available or not), pathogenicity(effect or causal relationship reported or not), geographical details from HapMap data (Yoruba, Japanese, Han, CEPH), change is in invariant region or not, model organisms details (studied in which model organism) and MeSH terms association (Fig 4A). The interaction level has 4 attributes with 32 properties and facilitates understanding the impact of variation on biological interactions. This level includes details on chemical bonds among macromolecules like salt-bridges, electrostatic interactions, hydrogen bonds, disulphide bonds etc, interactors (small molecule or bio polymer), genetic interactions (positive, neutral, negative or inequality interactions) and the complexes (protein-protein, protein-DNA, protein-RNA, protein-small molecule) as shown in Fig 4B. In all there are 48 attributes and 278 properties to capture the variation annotation across six levels and also fulfills the five-star vocabulary requirements by Bernard Vatant (http://bvatant.blogspot.fr/2012/02/is-your-linked-data-vocabulary-5-star_9588.htm).

### Fingerprinting Ontology Terms Using Binary Codes

**Fig 5:**
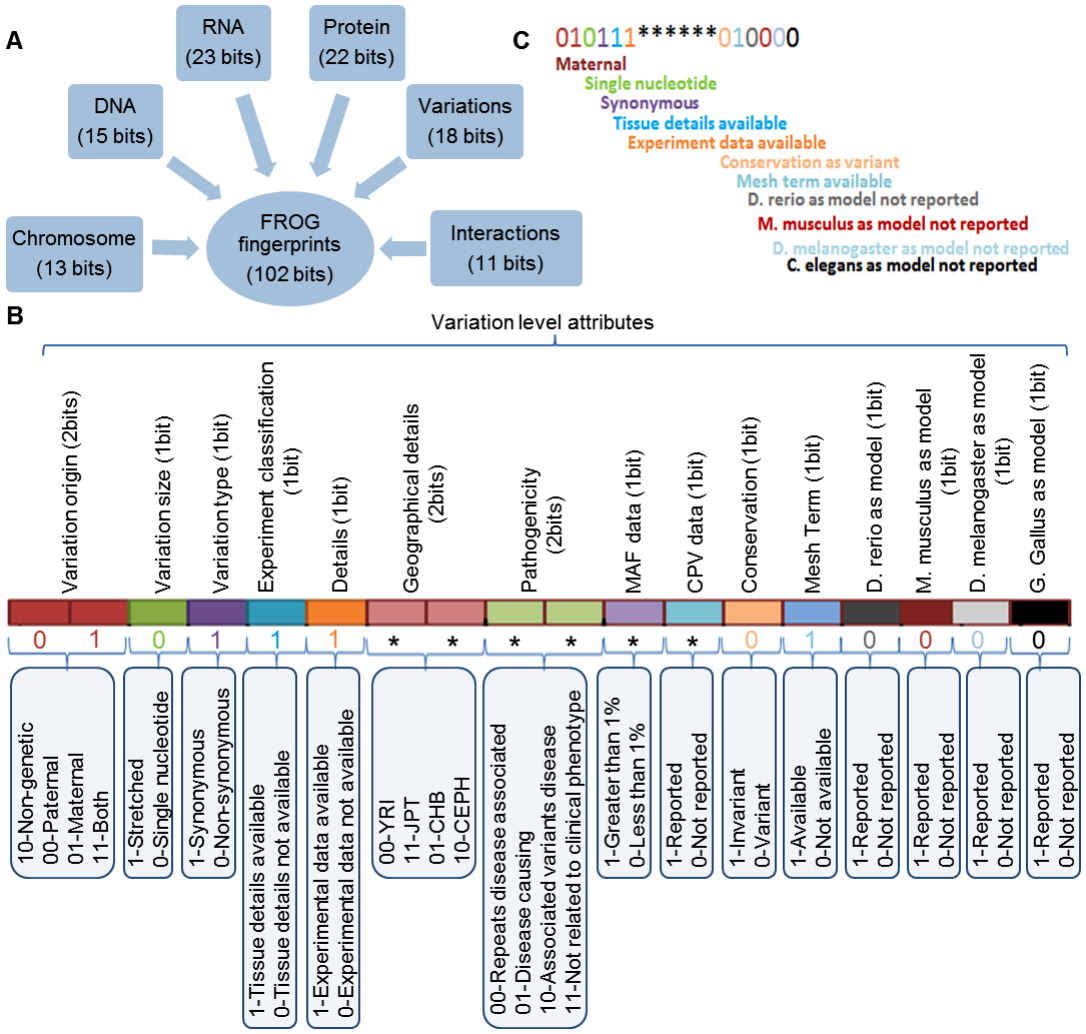
(A) Summary of the fingerprints designed to represent ontology terms in the six levels (B) As an example, the 15 attributes of the Variation level are listed along with their bit annotation (C) Example in Table 2 is explained with help of color coded bits.

As discussed above, FROG fingerprints comprise of a set of 102 binary bits representing 278 properties of genomic variation. These fingerprints are broadly divided into six levels including Chromosome level wherein 13 bits are used for representing 38 properties at chromosome level. Likewise, in DNA level 36 properties are represented using 15 bits, in RNA level 58 properties with 23 bits, Protein level represents 78 properties in 22 bits, variation with 36 properties in 18 bits and Interaction level with 32 properties using 11 bits (Fig 5A).

Table 2 shows description of fingerprinting of a variant from the sample set and the details of attributes and the fingerprint are shown in Fig 5B and 5C. The variant fingerprint starts with initial two bits tag to map the origin of variation (Maternal). Likewise, the third bit describes whether the variation has occurred on the stretch or single nucleic acid or amino acid in the DNA/RNA/protein, which is captured by 1 for stretch changes and 0 for single nucleotide changes. The fourth bit describes whether synonymous or non-synonymous change has occurred with values 1 and 0, respectively. The fifth bit indicates availability of tissue data and sixth bit indicates availability of experiment data. Likewise the subsequent bits store information on the geographic location as described in HapMap, with 00 for YRI, 11 for JPT, 01 for CHB and 10 for CEU. Pathogenicity is reported in two bits where 00 represents the repeats associated case, 01 the case for associated variations to cause the disease, 10 for the actual disease causing variations and 11 for the variations that is not related to pathogenicity. Minor allele frequency (MAF), if reported is given in single bit and similarly, the remaining bits indicates whether copy number variation (CNV) is reported or not, variation is in invariant region or non-invariant region, the associated MeSH terms and the model organisms. The details of the fingerprints and the combinations may be seen at FROG framework described in the next section.

**Table 2:** Example variant with assigned terms and fingerprints.

Column Name	Value	Description
Variant Id	531	Database (MitoLSDB) ID
Genomic position	m.10084T>C	mtDNA T>C change at 10084
Disease	diabetes- Angiopathy	Associated disease
Protein Name	ND3	Affected protein
Changed amino acid	p.I9T	Change of Isoleucine to Threonine at position 9 of protein.
DNA Level fingerprints	01011**********	First 3 bits (010): Transition
		4^th^ and 5^th^ bits (11): Transition (T to C)
Protein Level fingerprints	00*1101***************	1^st^ and 2^nd^ bits (00): Substitution
		4^th^, 5^th^,6^th^ and 7^th^ (1101): Pathogenecity as benign and tolerated predicted by Sift and Polyphen, respectively
Variation Level fingerprints	010111******010000	1^st^ and 2^nd^ bits (01): Maternal
		3^rd^ bit (0): Single nucleotide
		4^th^ bit (1): Synonymous
		5^th^ bit (1): Tissue details available
		6^th^ bit (1): Experimental data available
		13^th^ bit (0): Conservation as variant
		14^th^ bit (1): Mesh term available
		15-18^th^ (000): Model organism not reported

(*): Information not available in the database (MitoLSDB) to generate fingerprint for respective variant property.

### Web-based interface to FROG

**Fig 6:**
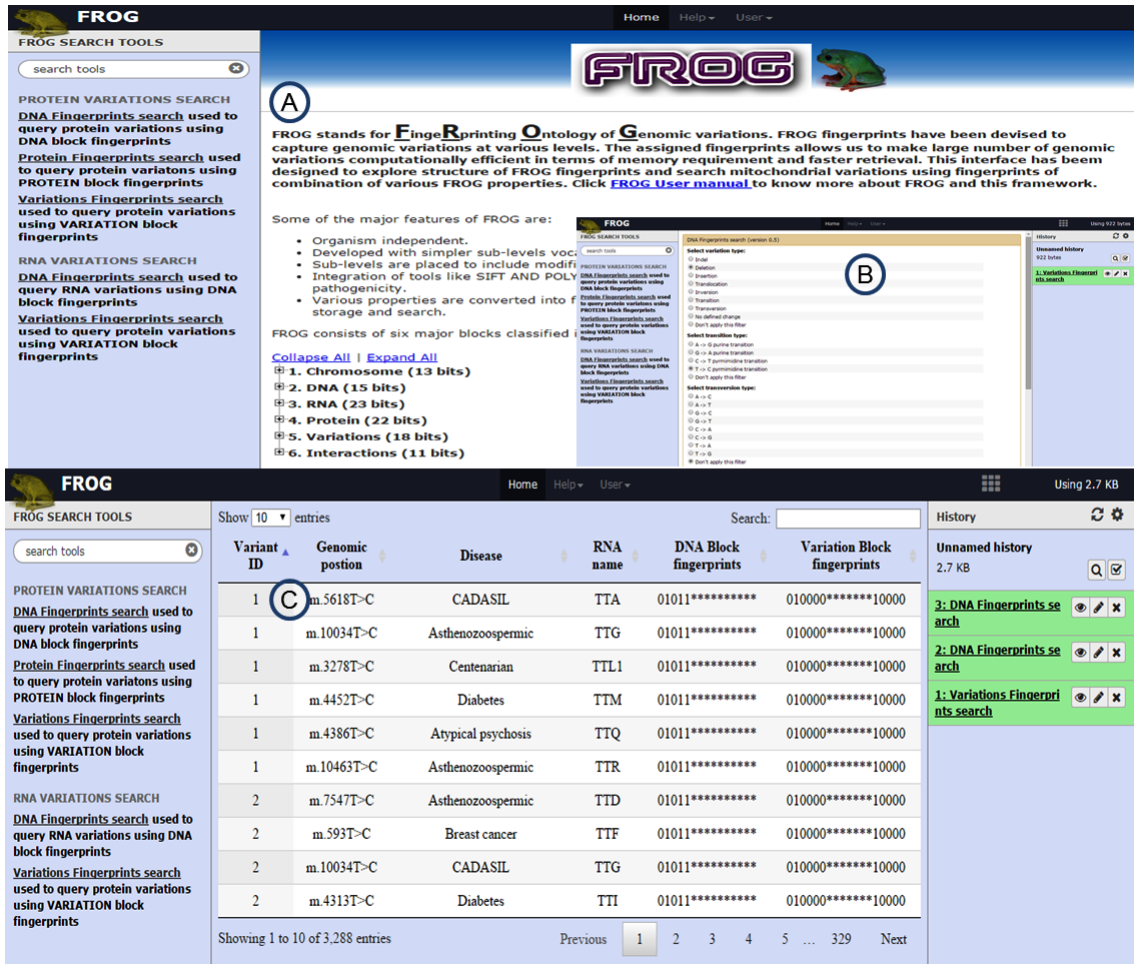
(A) The screenshot of FROG Interface displaying ontology terms and associated fingerprints (B) The search tool interface to query the DNA variation using ontology (C) Sample output of the search tool.

The rapidly increasing amount of genomic variations data poses a challenge for storage and searching complex queries efficiently. An example dataset of 29,241 RNA and 1,13,255 protein variants from MitoLSDB [18] have been used as a test case to generate binary fingerprints using FROG. These fingerprints are stored in a database wrapped by Galaxy framework [19]. The framework allows visualization of FROG ontologies terms and their associated fingerprints (Fig 6A). Within the framework, FROG search tools are available that streamline querying variations data using combinations of variation properties (Fig 6B and 6C). As an example, if one retrieves all protein variations caused by thymine to cytosine transition in DNA. This query can be performed using DNA fingerprints search tool provided under Protein variations search category by selecting transition type as T->C. If no other filters are applied, query results in ~21000 protein variations. These results can further be filtered for their association with a phenotype. The hierarchy of FROG fingerprints into different levels can also be visualized through framework, available at http://ab-openlab.csir.res.in/frog. FROG framework also offers a tool to generate fingerprints of different levels for user-supplied data. Thus, the interface facilitates understanding of the variation and the fingerprinting method with help of examples.

## Conclusion

It is imperative to make genomic variation easily understandable to non-ontology experts to ensure its wide spread implementation. The concept of fingerprinting in FROG is designed based on general understanding of functional impact of any variation. In addition to its simplicity, it is also developed for scalability in storage and search. A glossary is generated describing each level, attribute, property and associated bit score for clarity. Given that the variation properties are stored in form of bits (0s and 1s), FROG is language independent too, which is considered as a major bottleneck in ontology implementation globally. The properties, attributes and levels are semantically linked and provide effective data management for flexibility in adding additional properties. The concept can be elaborated as part of the semantic web initiative as has been implemented in case of UniProt RDF [22] and chem2bio–RDF [23]. Given that keyword searches applied to variable descriptions do not always provide accurate results due to syntactic and lexical complexities associated with the descriptions such as use of negation and synonyms [24], there is a need for common standard like FROG that bypass the language barrier for ontology sharing and implementation. In FROG, each level is modular and the levels are designed in such a way that it could allow further modifications in future like additions of new properties or attributes.

FROG offers an easy to understand system to capture different properties of genotype and phenotype data. Future versions of FROG will include extensive attributes and properties to capture clinical data which as of now is limited to MeSH terms. The system will be extended in form of binary fingerprints for computational scalability, language independence, organism and phenotype independence.
